# Undefined familial colorectal cancer and the role of pleiotropism in cancer susceptibility genes

**DOI:** 10.1007/s10689-016-9914-4

**Published:** 2016-06-29

**Authors:** Sara E. Dobbins, Peter Broderick, Daniel Chubb, Ben Kinnersley, Amy L. Sherborne, Richard S. Houlston

**Affiliations:** 1 Division of Genetics and Epidemiology, The Institute of Cancer Research, London, UK; 2 Division of Pathology, The Institute of Cancer Research, London, UK

**Keywords:** Cancer susceptibility, Colorectal, Pleiotropism, Familial colorectal cancer, Exome sequencing, Germline

## Abstract

Although family history is a major risk factor for colorectal cancer (CRC) a genetic diagnosis cannot be obtained in over 50 % of familial cases when screened for known CRC cancer susceptibility genes. The genetics of undefined-familial CRC is complex and recent studies have implied additional clinically actionable mutations for CRC in susceptibility genes for other cancers. To clarify the contribution of non-CRC susceptibility genes to undefined-familial CRC we conducted a mutational screen of 114 cancer susceptibility genes in 847 patients with early-onset undefined-familial CRC and 1609 controls by analysing high-coverage exome sequencing data. We implemented American College of Medical Genetics and Genomics standards and guidelines for assigning pathogenicity to variants. Globally across all 114 cancer susceptibility genes no statistically significant enrichment of likely pathogenic variants was shown (6.7 % cases 57/847, 5.3 % controls 85/1609; *P* = 0.15). Moreover there was no significant enrichment of mutations in genes such as *TP53* or *BRCA2* which have been proposed for clinical testing in CRC. In conclusion, while we identified genes that may be considered interesting candidates as determinants of CRC risk warranting further research, there is currently scant evidence to support a role for genes other than those responsible for established CRC syndromes in the clinical management of familial CRC.

**Electronic supplementary material:**

The online version of this article (doi:10.1007/s10689-016-9914-4) contains supplementary material, which is available to authorized users.

## Introduction

Family history is a major risk factor for colorectal cancer (CRC) with around 15 % of patients reporting having a first-degree relative affected with CRC [1]. Understanding the genetic basis of familial CRC risk is clinically relevant for discriminating between high- and low-risk groups; important not only in defining screening requirements and genetic counselling but increasingly for optimising chemotherapy [2].

Although Lynch syndrome and familial adenomatous polyposis (FAP), caused by inherited germline mismatch repair (MMR) and *APC* gene mutations respectively, contribute significantly to CRC a genetic diagnosis cannot be obtained in over 50 % of familial cases [3]. While Amsterdam positive families negative for MMR mutations have been labelled as Familial CRC Type X [4] this is merely a descriptive definition and the genetic basis of all forms of undefined-familial CRC is likely to be complex.

Many cancer susceptibility genes are pleiotropic (i.e. influence multiple types of malignancies) [5] and epidemiological studies have reported moderate increases in risk of CRC associated with a number of cancer susceptibility genes. It has been recently suggested that mutations in cancer susceptibility genes not normally considered primarily as determinants of CRC risk such as *BRCA2* [6] and *TP53* [7] contribute significantly to CRC and are of clinical utility.

To clarify the contribution of non-CRC cancer susceptibility genes to undefined-familial CRC we report a comprehensive mutational screen of 114 such genes in 847 patients systematically ascertained with early-onset undefined-familial CRC.

## Materials and methods

### Cancer susceptibility genes evaluated

We evaluated a set of 114 well established cancer susceptibility genes in which rare mutations have been documented to confer high or moderate risk of cancer [5].

### Subjects, sequencing and quality control

We report data on 857 unrelated cases of undefined-familial CRC that were negative for a mutation in a known cancer susceptibility genes for CRC. Specifically, there was no evidence of a likely pathogenic mutation in one of the known CRC genes—*APC*, *MLH1*, *SMAD4*, *BMPR1A*, *MUTYH*, *MSH2*, *MSH6*, *PMS2*, *POLE* or *POLD1*. The cases were derived from a previous whole exome sequencing (WES) gene discovery project based on 1028 familial cases (≥1 first-degree relative) with early-onset CRC (≤55 years) ascertained through the UK National Study of Colorectal Cancer Genetics [8, 9]. To determine the population prevalence of cancer susceptibility gene mutations we analysed WES data on 1644 healthy individuals (with no personal history of malignancy) from the UK 1958 Birth cohort (58BC [10]—974 from the ICR1000 dataset (EGAD00001001021) [11] and an additional 670 individuals all sequenced at The Institute of Cancer Research as per cases.

Full details of sample ascertainment, sequencing pipeline for these samples have been reported [9]. Briefly, samples with non-northern European ancestry, high levels of heterozygosity, sex discrepancy, poor call rate and contamination were excluded. We considered only canonical transcripts and for each variant, assumed the most deleterious predicted effect for each Ensembl transcript according to Variant Effect Predictor [12]. To identify false positives we adopted an automated approach imposing: GQ ≥ 30, for a heterozygous call an alternate depth ≥3 and χ^2^ < 10.83 (i.e. *P* > 0.0001) for the observed versus expected distribution of alternate/reference alleles (alt-ref-ratio), UCSC alignability (100 bp window size) = 1, not in simple repeat, Hardy–Weinberg Equilibrium (HWE) test (*P* > 1.0 × 10^−8^) in cases and controls and an overall call rate ≥75 % in both cases and controls. We evaluated the fidelity of sequencing in 1332 samples which had also been genotyped using Illumina HumanExome-12v1_A Beadchip arrays (Illumina, San Diego, CA, USA). Specificity and sensitivity of across all alleles with MAF < 0.05 was >99.99 % and 78.4 % respectively for filtered variants.

### Interpretation of variant pathogenicity

We implemented the American College of Medical Genetics and Genomics (ACMG) standards and guidelines for determining the pathogenicity of variants [13]. ACMG definitions are contingent on the population frequencies of variants. Here we utilised frequency information from the non-Finnish European Exome Aggregation Consortium (NFE-ExAC), excluding The Cancer Genome Atlas (TCGA) samples when appropriate (nonTCGA-NFE-ExAC). For assigning pathogenicity of novel missense mutations ACMG requires the rate of benign missense variation to be determined in genes and functional domains (PM1 and PP2). ACMG defines mutations as benign if seen in >5 % of the population (BA1). We exploited this definition, counting the frequency of missense mutations in our dataset with NFE-ExAC frequency >5 %, to quantify the rate of benign missense variation. When normalised for protein length, none of the cancer susceptibility genes evaluated in this study fell outside of two standard deviations from the average mutational rate of 1.2 mutations per 1000 amino acids (<6.6). ACMG also requires novel mutations to conform to established mechanisms of action for each gene, therefore established mechanisms were determined by mining pathogenic/likely pathogenic (P/LP) mutations for any disease in ClinVar (NFE-ExAC < 1 %) and combined with annotations from the expertly curated Cosmic Gene Census [14] and where available relevant literature.

Definition of pathogenic/likely pathogenic (P/LP) variants: We required all variants to be rare with a nonTCGA-NFE-ExAC frequency <0.01 % for dominantly acting genes or <0.5 % for genes with evidence of recessive action. We automatically included variants marked as “pathogenic” or “likely pathogenic” in ClinVar which met the above frequency conditions and where ClinVar annotations were not conflicted. Novel loss of function (LOF) and splice site variants met the criteria for likely pathogenic only when the mechanism of action (*e*.*g*. splice site) was established for that gene. Only canonical transcripts were considered. LOF variants at the extreme 3′ end of the gene were excluded from analysis (final coding 5 % [15]). Missense mutations were included where the same amino acid change was observed as a previously established P/LP variant regardless of nucleotide change.

Definition of novel likely pathogenic (LP) missense set: We also defined a set of novel (not documented in Clinvar) likely pathogenic missense mutations requiring, in addition to the criteria outlined above: (1) a consensus that the mutation is deleterious in a minimum of 6/8 computational tools calculated via ANNOVAR [16] (satisfying PP3 criteria; SIFT, Polyphen-pp2hvar, LRT, MutationTaster, MutationAssessor, FATHMM, RadialSVM and LR) (2) that the variant is in a gene with a low rate of benign missense variation (PP2) (3) located in PFAM domain with documented P/LP variants with no benign variation (PM1) and (4) for dominantly acting variants only: absent from the nonTCGA-NFE-ExAC population (PM2).

*P* values, where reported, were calculated using a two-sided Fisher’s exact test in R [17].

## Results and discussion

Overall 6.7 % of the undefined-familial CRC cases (57/847) and 5.3 % of the controls (85/1609) were identified as being a carrier of a P/LP mutation in one of the 114 cancer susceptibility genes surveyed (Table 1; Supplementary Table 2). Globally the difference is not statistically significant implying that pleiotropic effects across cancer susceptibility genes are not widespread with respect to CRC and certainly rare and/or not highly penetrant. Furthermore, after correcting for multiple testing, no individual gene was significantly mutated in undefined-familial CRC cases. This does not preclude the possibility that some of the mutations we have identified are causal, but does place bounds on their prevalence and clinical utility.

**Table 1:** Case/control statistics for cancer susceptibility genes

Gene	P/LP variant set		*P*	Novel LP missense set	
	Case	Control		Case	Control
*FLCN*	3	0	0.04	0	0
*ERCC3*	3	1	0.12	0	0
*BLM*	3	1	0.12	1	0
*BRCA2*	5	3	0.14	1	0
*ERCC4*	4	2	0.19	0	0
*BRIP1*	6	5	0.21	0	1
*RAD51D*	2	1	0.28	0	0
*ERCC5*	1	0	0.35	1	0
*POLH*	1	0	0.35	6	6
*MEN1*	1	0	0.35	1	1
*DDB2*	1	0	0.35	0	0
*TGFBR1*	1	0	0.35	0	0
*CYLD*	1	0	0.35	0	0
*BUB1B*	1	0	0.35	0	0
*NF1*	1	0	0.35	0	0
*TP53*	1	0	0.35	0	1
*EXT2*	1	0	0.35	0	2
*BRCA1*	4	5	0.51	0	0
*NBN*	2	2	0.61	0	0
*CHEK2*	1	4	0.66	1	0
*FAH*	1	5	0.67	3	4
*ATM*	4	8	1	0	0
*RECQL4*	3	6	1	0	0
*FANCC*	2	4	1	0	0
*ERCC2*	2	3	1	0	0
*COL7A1*	1	3	1	13	22
*SERPINA1*	1	2	1	0	1
*SLC25A13*	1	2	1	0	0
*TRIM37*	1	1	1	0	0
*FANCG*	1	1	1	0	0
*PALB2*	1	1	1	0	0

Any gene with at least one case P/LP mutation is included and ranked by Fisher’s exact *P* value. Some individuals carry variants in more than one gene

Three cases (0.3 %) were identified as being carriers of P/LP mutations in the folliculin gene *FLCN*: a stop gain (p.Ser386Ter), splice donor (c.1432 + 1G>A) and a frameshift variant (p.Glu297AlafsTer25) catalogued by ClinVar as pathogenic (Table 2). These mutations are extremely rare with only the frameshift variant present in a single sample in nonTCGA-NFE-ExAC. *FLCN* is a highly conserved gene and recent computational methods have predicted *FLCN* to be intolerant to LOF variants (analysis of protein-coding genetic variation in 60,706 humans, Lek et al. preprint). While mutations in *FLCN* cause Birt-Hogg-Dube syndrome (BHD) [18], and are seen in 5 % of familial renal cell cancer patients suggesting a role in cancer predisposition [19], none of the gene carriers we identified had a personal or family history of renal cancer. Although recent data has been conflicted as to whether there is an increased incidence of CRC associated with BHD, Nahorski et al. [20] have reported *FLCN* deactivation contributes to colorectal tumourigenesis with somatic frameshift mutations being identifiable in 23 % of microsatellite instable CRC.

**Table 2:** Summary information for the mutations observed in the cancer susceptibility genes: *FLCN*, TC-NER genes, *BLM* and *BRCA1/2*

Gene	cDNA change	Protein change	Consequence	Case	Control	ExAC^a^ count	ClinVar
*FLCN*	c.1432 + 1G>A		SD	1	0	0	–
	c.1157C>G	p.Ser386Ter	SG	1	0	0	–
	c.890_893delAAAG	p.Glu297AlafsTer25	FS	1	0	1	P
*ERCC2*	c.2138G>A	p.Gly713Asp	M	0	1	0	P
	c.1827delC	p.Phe610LeufsTer99	FS	1	0	1	–
	c.1381C>G	p.Leu461Val	M	1	2	31	P
*ERCC3*	c.1757delA	p.Gln586ArgfsTer25	FS	2	0	18	–
	c.1421dupA	p.Asp474GlufsTer2	FS	1	0	6	–
	c.325C>T	p.Arg109Ter	SG	0	1	42	–
*ERCC4*	c.458G>A	p.Arg153His	M	1	0	1	P
	c.1399delA	p.Arg468AspfsTer25	FS	1^e^	0	0	–
	c.2395C>T	p.Arg799Trp	M	2	2	35	P
*ERCC5*	c.274A>T^b^	p.Arg92Trp	M	1	0	1	–
	c.1207_1208insTGTGTGC	p.Gly406ValfsTer5	FS	1	0	0	–
*ERCC6* ^c^	c.2167C>T	p.Gln723Ter	SG	2	1	4	P
	c.1958A>G^b^	p.Asn653Ser	M	1	0	0	–
	c.1780G>A^b^	p.Val594Met	M	0	1	0	–
	c.2093dupG	p.Thr699HisfsTer61	FS	1	0	0	–
*XPA*	c.338_339delTG	p.Met113ArgfsTer9	FS	0	1	1	–
*BLM*	c.1081_1082delTG	p.Cys361Ter	FS	1	0	0	–
	c.1933C>T	p.Gln645Ter	SG	1	0	4	–
	c.2422C>T^b^	p.Arg808Cys	M	1	0	7	–
	c.2695C>T	p.Arg899Ter	SG	1	1	6	LP;P
*BRCA1*	c.5186C>A	p.Ala1729Glu	M	1	0	2	P
	c.5158C>T	p.Arg1720Trp	M	0	1	0	P
	c.3756_3759delGTCT	p.Ser1253ArgfsTer10	FS	2	1	0	LP;P
	c.3228_3229delAG	p.Gly1077AlafsTer8	FS	1^d^	0	0	LP;P
	c.2194G>T	p.Glu732Ter	SG	0	1	0	P
	c.1849_1850delAC	p.Thr617GlnfsTer7	FS	0	1	0	–
	c.246delT	p.Val83LeufsTer5	FS	0	1	0	–
*BRCA2*	c.3158T>G	p.Leu1053Ter	SG	0	1	0	P
	c.3680_3681delTG	p.Leu1227GlnfsTer5	FS	1	0	0	P
	c.3689delC	p.Ser1230LeufsTer9	FS	1^d^	0	0	P
	c.5682C>A	p.Tyr1894Ter	SG	1	0	0	P
	c.5946delT	p.Ser1982ArgfsTer22	FS	0	1	21	P;RF
	c.6275_6276delTT	p.Leu2092ProfsTer7	FS	1	0	1	P
	c.6535_6536insA	p.Val2179AspfsTer10	FS	1	0	1	–
	c.7933A>G^b^	p.Arg2645Gly	M	1	0	0	–
	c.9054_9055delTA	p.Ser3018ArgfsTer3	FS	0	1	0	P

Conseqeuence: *SD* splice donor, *SG* stop gain, *FS* frameshift, *M* missense. Clinvar: *P* pathogenic, *LP* likely pathogenic, *RF* risk factor

^a^NonTCGA-NFE-ExAC count

^b^LP Missense variant

^c^ *ERCC6* not in cancer susceptibility gene set but included for information

^d^Case has FS mutations affecting *BRCA1* and *BRCA2*

^e^Case has SG in *PALB2* (Tyr1183Ter; Exac = 1) although Clinvar pathogenic affects only final 1 % of protein

A further three cases had P/LP frameshift mutations in the nucleotide excision repair (NER) gene *ERCC3*; two cases with p.Gln586ArgfsTer25 and a single sample with p.Asp474GlufsTer2 (Table 2). We observe a LP mutation in a single control: p.Arg109Ter. *ERCC3* forms a subunit of the basal transcription factor 2 (TFIIH, Table 1) and is associated with Xeroderma pigmentosum B [21], Cockayne’s syndrome [22] and trichothiodystrophy [23]. A variant in the related NER protein ERCC6 was recently suggested as a candidate for familial CRC following exome sequencing with functional data supporting a reduction in capacity for repairing DNA double strand breaks [24]. In transcription coupled (TC)-NER, blockage of transcribing RNA Polymerase II (RNA-Pol II) on the damaged DNA template is thought to initiate the repair reaction in a process that requires ERCC6 in combination with ERCC2 (TFIIH subunit), ERCC3 (TFIIH subunit), ERCC1-ERCC4 (XPF), ERCC5 (XPG), ERCC8 (CSA) and XPA. We observed P/LP mutations in six of these genes in total identifying 13 in cases and eight in controls (Table 2, 1.5 vs 0.5 %, *P* = 0.011; 15 vs 9 including LP missense, *P* = 0.008). We identify two patients with first degree relatives with malignant melanoma of the skin (*ERCC4* p.Arg468AspfsTer25 and *ERCC6* p.Thr699HisfsTer61). With the exception of *ERCC6* thus far mutations of the NER genes have not been implicated as risk factors for CRC. It is however noteworthy that *ERRC1* expression has been shown to be reduced by 84–100 % in CRC [25, 26].

Three cases and one control were found to harbour rare novel LOF mutations in the recessively acting Bloom syndrome gene *BLM* (Table 2): one case with the frameshift mutation p.Cys361Ter, another with the stop gain mutation p.Gln645Ter and an additional case and control both having the stop gain mutation p.Arg899Ter catalogued as pathogenic and likely pathogenic in ClinVar for hereditary cancer predisposition syndrome and Bloom’s respectively. An additional rare missense mutation meeting ACMG guidelines for likely pathogenic variants was identified in the cases (p.Arg808Cys, no such variants in controls). While the family histories of these samples are varied, only one Bloom’s related malignancy (myeloma) was reported in the mother of an affected individual where the father was diagnosed with CRC. While our results could be considered to support a possible role for *BLM* in CRC risk, we do not observe as high a frequency of P/LP mutations as a recent study which found enrichment in early-onset CRC patients with deleterious *BLM* mutations (1.6 % of patients and 0.02 % controls [27]).

With respect to *BRCA1*/*2* mutations we observed a range of mutation types including ten frameshift (6 cases, 5 controls) three stop gained (1 case, 2 controls) and two missense (1 case, 1 control) with the majority of variants (12/15) documented as pathogenic by ClinVar (0.9 % vs 0.5 %, *P* = 0.20, Table 2). Whilst 5/8 of cases had a family history of breast and/or ovarian cancer, in three cases CRC was observed in the father with the *BRCA* associated cancer observed in the mother. Of these three patients, one carried *BRCA1*:p.Gly1077AlafsTer8 and *BRCA2*:p.Ser1230LeufsTer9, both catalogued as pathogenic in ClinVar and absent in nonTCGA-NFE-ExAC.

Of the other cancer susceptibility genes recently implicated in CRC, including *WRN* [24], *SMARCA4* [28], *AXIN2* [29, 30] and *TP53* [7], we only identified a single case with a mutation: *TP53* p.Glu68Ter. This case, a male aged 48 at diagnosis, had no personal history of other cancers or a family history of any Li-Fraumeni associated malignancy. Our results do not support the recent assertion of a clinically important role for *TP53* in CRC [7]. It is however noteworthy that the *TP53* mutations reported by Yurgelun et al. [7] were all (bar one) predicted benign missense changes and no reference to gene burden in a comparison with controls was performed.

## Conclusion

In a large number of patients with familial CRC no alteration in any known CRC susceptibility gene can be identified. An explanation of their susceptibility is a priority in order to offer accurate genetic counselling and determine appropriate screening and/or treatment. Many cancer susceptibility genes have pleiotropic effects increasing the risk of a spectrum of cancers to varying degrees [5]. Hence the suggestion that non-CRC cancer susceptibility genes contribute to familial CRC is an attractive proposition.

However the risks for the minor type of cancer are in general modest with, for example, studies suggesting a 20–60 % increase in risk associated with *BRCA2* mutations and cancers outside of breast and ovarian [6]. This is in contrast to mutations in genes such as *TP53* that are typified by a constellation of cancers in the same family. In addition to the phenotypic variability associated with the classical dominantly acting cancer susceptibility genes there is evidence of increased cancer risk in carriers of recessive cancer syndrome mutations; exemplified by heterozygous *ATM* mutations associated with a two-fold elevated breast cancer risk [31]. The magnitude of these effects are therefore insufficient to result in families segregating only the minor tumour. While this means cancer susceptibility gene pleiotropism will not significantly account for undefined-familial CRC families per se such effects have the potential to impact on the overall burden of CRC.

Even accepting the potential inflation introduced through using cancer free controls, when considering all 114 cancer susceptibility genes, we did not observe a significant difference in frequency of pathogenic mutations between cases and controls. Although only nominally significant we did identify P/LP mutations in a number of interesting candidate genes including *FLCN*, *BLM*, *ERCC*-genes and *BRCA1/2* as possible determinants of CRC risk.

Accurately ascribing pathogenicity to variants is a key challenge in interpreting sequencing data and we must be cognisant of variation that is disregarded or missing such as splice region or copy number variation. While our estimated frequency of P/LP mutations broadly fits with epidemiological estimates, it is likely that some of the cancer susceptibility genes included in this analysis are able to tolerate apparent LOF variants. Indeed we observed some over-representation of *BRIP1* P/LP mutations amongst cases (6 cases, 5 controls) and a study by Seal et al. [32] had reported rare truncating mutations in *BRIP1* with increased breast cancer risk. However a large replication effort of the most common truncating variant found no evidence to support an association between *BRIP1* with breast cancer [33].

While we identified genes that may be considered interesting candidates for further research our inability to replicate other recent studies highlights the caution required when interpreting such research. Additional much larger data sets, familial studies and/or functional follow up would be required to confirm the role and scope of cancer susceptibility genes outside of those already clearly established with heritable CRC syndromes. However, compared to contemporaneous research efforts, a major strength of our study is its size enabling us to explore the maximum likely impact of non-CRC cancer susceptibility genes to undefined-familial CRC. In conclusion there is currently scant evidence to support a role for genes other than those responsible for established CRC syndromes in the clinical management of CRC patients. While testing for such genes has no immediate clinical utility, the accumulation of such data, in combination with functional studies and familial segregation, has the potential to robustly determine the role of these genes in CRC aetiology. Furthermore, as the cost of high throughput sequencing continues to reduce, such efforts may become economically justifiable.

## Electronic supplementary material

Below is the link to the electronic supplementary material.

Supplementary material 1 (DOCX 98 kb)
