# Supplementary material for: Undefined familial colorectal cancer and the role of pleiotropism in cancer susceptibility genes
Source: Fam Cancer. 2016 Jun 29;15(4):593–9. doi: 10.1007/s10689-016-9914-4 (PMC5010824; doi:10.1007/s10689-016-9914-4)
Supplement: Supplementary file 1 — Supplementary material 1 (DOCX 98 kb) [file 10689_2016_9914_MOESM1_ESM.docx]

**Supplementary Table 1: Cancer Susceptibility Genes**. Data extracted from: *Rahman N. Realizing the promise of cancer predisposition genes. Nature 2014;505(7483):302-8*.

| ***Gene*** | **Gene Name** | **Chromosome location** | **Cancer syndrome(s)** | **Major associated tumor types** | **Mode of inheritance** |
| --- | --- | --- | --- | --- | --- |
| *ABCB11* | ATP-binding cassette, sub-family B (MDR/TAP), member 11 | 2q31.1 | Progressive familial intrahepatic cholestasis | Hepatocellular carcinoma Cholangiocarcinoma | autosomal recessive |
| *ALK* | anaplastic lymphoma receptor tyrosine kinase | 2p23 |  | Neuroblastoma | autosomal dominant |
| *APC* | adenomatous polyposis coli | 5q21 | Familial adenomatous polyposis (FAP) | Colorectal cancer  Hepatoblastoma Desmoid tumor | autosomal dominant |
| *ATM* | ataxia telangiectasia mutated | 11q22.3 | Ataxia-Telangiectasia (biallelic mutations) | Biallelic mutations: Lymphoid hematological malignancy (leukemia, lymphoma)  Monoallelic mutations:  Breast cancer | autosomal recessive  autosomal dominant |
| *AXIN2* | axin 2 | 17q24.1 | oligodentia-colorectal cancer syndrome | Colorectal cancer | autosomal dominant |
| *BAP1* | BRCA1 associated protein-1 (ubiquitin carboxy-terminal hydrolase) | 3p21.31-p21.2 |  | Melanoma (cutaneous, uveal) Mesothelioma Meningioma Lung cancer (adenocarcinoma) | autosomal dominant |
| *BLM* | Bloom syndrome, RecQ helicase-like | 15q26.1 | Bloom syndrome | Lymphoma and ALL hematological malignancy Myeloid hematological malignancy Squamous cell carcinoma, scc gastic, colorectal cancers | autosomal recessive |
| *BMPR1A* | bone morphogenetic protein receptor, type IA | 10q22.3 | Juvenile polyposis syndrome | Colorectal cancer, gastric cancer, hamartoma | autosomal dominant |
| *BRCA1* | breast cancer 1, early onset | 17q21 | Hereditary breast-ovarian cancer | Breast cancer Ovarian cancer | autosomal dominant |
| *BRCA2* | breast cancer 2, early onset | 13q12 | Hereditary breast-ovarian cancerFanconi anaemia (D1) (biallelic mutations) | Biallelic mutations:Myeloid hematological malignancy (MedulloblastomaWilms tumorMonoallelic mutations:Breast cancerOvarian cancerProstate cancerPancreas cancer | autosomal recessive autosomal dominant |
| *BRIP1* | BRCA1 interacting protein C-terminal helicase 1 | 17q22 | Fanconi anaemia (J) (biallelic mutations) | Biallelic mutations: Myeloid hematological malignancy  Squamous cell carcinoma (head and neck, esophagus, genital tract) Monoallelic mutations: Breast cancer Ovarian cancer | autosomal recessive  autosomal dominant |
| *BUB1B* | budding uninhibited by benzimidazoles 1 homolog beta (yeast) | 15q15 | Mosaic variegated aneuploidy Syndrome | Wilms Tumor  Rhabdomyosarcoma Myeloid hematological malignancy | autosomal recessive |
| *CBL* | Cbl proto-oncogene, E3 ubiquitin protein ligase | 11q23 | Noonan syndrome | JMML | autosomal dominant |
| *CDC73* | cell division cycle 73, Paf1/RNA polymerase II complex component, homolog (S. cerevisiae) | 1q25 | Hyperparathyroidism-jaw tumor syndrome | Parathyroid cancer Ossifying fibroma (bone) | autosomal dominant |
| *CDH1* | cadherin 1, type 1, E-cadherin (epithelial) | 16q22.1 | Hereditary diffuse gastric cancer | Breast cancer (lobular) Gastric cancer (diffuse) | autosomal dominant |
| *CDK4* | cyclin-dependent kinase 4 | 12q14 |  | Melanoma | autosomal dominant |
| *CDKN1B* | cyclin-dependent kinase inhibitor 1B (p27, Kip1) | 12p.13.1 |  | Thyroid cancer, Pituitary adenoma | autosomal recessive autosomal dominant |
| *CDKN2A* | cyclin-dependent kinase inhibitor 2A | 9p21 |  | Melanoma [p16 and p14ARF] Pancreas cancer [p16 ] Astrocytoma [p14ARF ] | autosomal dominant |
| *CEBPA* | CCAAT/enhancer binding protein (C/EBP), alpha | 19q13.1 |  | Myeloid hematological malignancy | autosomal dominant |
| *CHEK2* | checkpoint kinase 2 | 22q12.1 |  | Breast cancer | autosomal dominant |
| *COL7A1* | collagen, type VII, alpha 1 | 3p21.31 | Epidermolysis bullosa | Squamous cell carcinmona (skin) | autosomal recessive  autosomal dominant |
| *CYLD* | cylindromatosis (turban tumor syndrome) | 16q12.1 | Brooke-Spiegler syndrome | Cylindroma spiroadenocarcinoma Basal cell carcinoma | autosomal dominant |
| *DDB2* | damage-specific DNA binding protein 2, 48kDa | 11p12 | Xeroderma Pigmentosum (E) | Basal cell carcinoma Squamous cell carcinoma Melanoma | autosomal dominant |
| *DICER1* | dicer 1, ribonuclease type III | 14q32.13 | DICER1 syndrome | Pleuropulmonary blastoma Cystic nephroma  Ovarian sex cord tumor | autosomal dominant |
| *DIS3L2* | DIS3 mitotic control homolog (S. cerevisiae)-like 2 | 2q37.1 | Perlman syndrome | Wilms tumor | autosomal recessive |
| *DKC1* | dyskeratosis congenita 1, dyskerin | Xq28 | Dyskeratosis congenita | acute myeloid leukemia Squamous cell carcinoma (head + neck, anorectal) | X-linked recessive |
| *DOCK8* | dedicator of cytokinesis 8 | 9p24.3 | HyperIgE syndrome | Squamous cell carcinoma Lymphoma | autosomal recessive |
| *EGFR* | epidermal growth factor receptor | 7p12 |  | Non-small cell lung cancer | autosomal dominant |
| *ELANE* | elastase, neutrophil expressed | 19p13.3 | Severe congenital neutropenia | Leukemia | autosomal dominant |
| *ERCC2* | excision repair cross-complementing rodent repair deficiency, complementation group 2 | 19q13.3 | Xeroderma pigmentosum (D) | Basal cell carcinomaSquamous cell carcinomaMelanoma | autosomal recessive |
| *ERCC3* | excision repair cross-complementing rodent repair deficiency, complementation group 3 | 2q21 | Xeroderma pigmentosum (B) | Basal cell carcinoma Squamous cell carcinoma Melanoma | autosomal recessive |
| *ERCC4* | excision repair cross-complementing rodent repair deficiency, complementation group 4 | 16p13.12 | Xeroderma pigmentosum (F) Fanconi anaemia (Q) | Basal cell carcinoma Squamous cell carcinoma Melanoma | autosomal recessive |
| *ERCC5* | excision repair cross-complementing rodent repair deficiency, complementation group 5 | 13q33 | Xeroderma pigmentosum (G) | Basal cell carcinoma Squamous cell carcinoma Melanoma | autosomal recessive |
| *EXT1* | exostosin 1 | 8q24.11 |  | Chondrosarcoma | autosomal dominant |
| *EXT2* | exostosin 2 | 11p12-p11 |  | Chondrosarcoma | autosomal dominant |
| *FAH* | fumarylacetoacetate hydrolase (fumarylacetoacetase) | 15q25.1 | Tyrosinemia | Hepatocellular carcinoma | autosomal recessive |
| *FANCA* | Fanconi anemia, complementation group A | 16q24.3 | Fanconi anaemia (A) | Myeloid hematological malignancy Squamous cell carcinoma (head and neck, esophagus, genital tract) | autosomal recessive |
| *FANCC* | Fanconi anemia, complementation group C | 9q22.3 | Fanconi anaemia (C) | Myeloid hematological malignancy Squamous cell carcinoma (head and neck, esophagus, genital tract) | autosomal recessive |
| *FANCG* | Fanconi anemia, complementation group G | 9p13 | Fanconi anaemia (G) | Myeloid hematological malignancy Squamous cell carcinoma (head and neck, esophagus, genital tract) | autosomal recessive |
| *FH* | fumarate hydratase | 1q42.1 | Hereditary leiomyomatosis and renal cell cancer (HLRCC) | Renal cell cancer Leiomyosarcoma (uterus) | autosomal recessive  autosomal dominant |
| *FLCN* | folliculin | 17p11.2 | Birt-Hogg-Dube syndrome | Renal cell cancer Oncocytoma | autosomal dominant |
| *GATA2* | GATA binding protein 2 | 3q21.3 | Emberger MonoMAC syndrome | Myeloid hematological malignancy | autosomal dominant |
| *GBA* | glucosidase, beta, acid | 1q21 | Gauchers type 1 | Myeloma Lymphoma Hepatocellular carcinoma | autosomal recessive |
| *GJB2* | gap junction protein, beta 2, 26kDa | 13q12.11 | Keratosis-icthyosis-deafness syndrome (KID) | Squamous cell carcinoma | autosomal dominant |
| *GPC3* | glypican 3 | Xq26.1 | Simpson-Golabi-Behmel syndrome | Wilms tumor Hepatoblastoma, hepatocellular carcinoma Neuroblastoma Gonadoblastoma | X-linked recessive |
| *HFE* | hemochromatosis | 6p22.2 | Haemochromatosis | Hepatocellular carcinoma Cholangiocarcinoma | autosomal recessive |
| *HMBS* | hydroxymethylbilane synthase | 11q23.3 | Porphyria (AI) | hepatocellular carcinoma | autosomal dominant |
| *HRAS* | v-Ha-ras Harvey rat sarcoma viral oncogene homolog | 11p15.5 | Costello syndrome | Rhabdomyosarcoma Neuroblastoma Transitional cell carcinoma (bladder) | autosomal dominant |
| *ITK* | IL2-inducible T-cell kinase | 5q33.3 | Lymphoproliferative syndrome 1 | Hodgkins lymphoma | autosomal recessive |
| *KIT* | v-kit Hardy-Zuckerman 4 feline sarcoma viral oncogene homolog | 4q12 |  | Gastro-Intestinal Stromal Tumor | autosomal dominant |
| *MAX* | MYC associated factor X | 14q23 | Familial paraganglioma-pheochromocytoma syndrome | Paraganglioma Pheochromocytoma | autosomal dominant |
| *MEN1* | multiple endocrine neoplasia I | 11q13 | Multiple endocrine neoplasia Type 1 | Parathyroid, pituitary adenoma  Neuroendocrine tumor Carcinoid tumor Adrenocortical carcinoma | autosomal dominant |
| *MET* | met proto-oncogene (hepatocyte growth factor receptor) | 7q31 |  | Renal cell cancer (papillary carcinoma) | autosomal dominant |
| *MLH1* | mutL homolog 1, colon cancer, nonpolyposis type 2 (E. coli) | 3p21.3 | MMR deficiency syndrome (biallelic mutations) Lynch syndrome / Hereditary Non-Polyposis Colon Cancer (monoallelic mutations) | Biallelic mutations: Brain tumors Hematological malignancy Embryonal tumors Monoallelic mutations: Colorectal cancer Endometrial cancer Ovarian cancer | autosomal recessive  autosomal dominant |
| *MSH2* | mutS homolog 2, colon cancer, nonpolyposis type 1 (E. coli) | 2p21 | MMR deficiency syndrome (biallelic mutations) Lynch syndrome / Hereditary Non-Polyposis Colon Cancer (monoallelic mutations) | Biallelic mutations: Brain tumors Hematological malignancy Embryonal tumors Monoallelic mutations: Colorectal cancer Endometrial cancer Ovarian cancer Sebaceous adenoma, carcinoma, epithelioma | autosomal recessive  autosomal dominant |
| *MSH6* | mutS homolog 6 (E. coli) | 2p16 | MMR deficiency syndrome (biallelic mutations) Lynch syndrome / Hereditary Non-Polyposis Colon Cancer (monoallelic mutations) | Biallelic mutations: Brain tumors Hematological malignancy Embryonal tumors Monoallelic mutations: Colorectal cancer Endometrial cancer Ovarian cancer | autosomal recessive  autosomal dominant |
| *MTAP* | methylthioadenosine phosphorylase | 9p21.3 | Diaphyseal medullary stenosis with malignant fibrous histiocytoma (DMS-MFH) | malignant fibrous histiocytoma (sarcoma) | autosomal dominant |
| *MUTYH* | mutY homolog (E. coli) | 1p34.1 |  | Colorectal cancer | autosomal recessive |
| *NBN* | nibrin | 8q21 | Nijmegen breakage syndrome | Lymphoma  Medulloblastoma Glioma Rhabdomyosarcoma | autosomal recessive |
| *NF1* | neurofibromin 1 | 17q12 | Neurofibromatosis type 1 | Glioma Malignant peripheral nerve sheath tumor | autosomal dominant |
| *NF2* | neurofibromin 2 (merlin) | 22q12.2 | Neurofibromatosis type 2 | Vestibular schwannoma Meningioma Ependymoma | autosomal dominant |
| *PALB2* | partner and localizer of BRCA2 | 16p12.1 | Fanconi anaemia (N) (biallelic mutations) | Biallelic mutations: Myeloid hematological malignancy  Medulloblastoma Neuroblastoma Wilms tumor Monoallelic mutations: Breast cancer Pancreas cancer | autosomal recessive  autosomal dominant |
| *PDGFRA* | platelet-derived growth factor receptor, alpha polypeptide | 4q12 |  | Gastro-Intestinal Stromal Tumor | autosomal dominant |
| *PHOX2B* | paired-like homeobox 2b | 4p12 |  | Neuroblastoma | autosomal dominant |
| *PMS2* | PMS2 postmeiotic segregation increased 2 (S. cerevisiae) | 7p22 | MMR deficiency syndrome (biallelic mutations) Lynch syndrome / Hereditary Non-Polyposis Colon Cancer (monoallelic mutations) | Biallelic mutations: Brain tumors  Hematological malignancy Supratentorial primitive neuroectodermal tumors Monoallelic mutations: Colorectal cancer Endometrial cancer Ovarian cancer | autosomal recessive  autosomal dominant |
| *POLD1* | polymerase (DNA directed), delta 1, catalytic subunit | 19q13.33 | PPAP (polymerase proofreading associated polyposis) | Colorectal cancer Endometrial cancer | autosomal dominant |
| *POLE* | polymerase (DNA directed), epsilon, catalytic subunit | 12q24.33 | PPAP (polymerase proofreading associated polyposis) | Colorectal cancer | autosomal recessive  autosomal dominant |
| *POLH* | polymerase (DNA directed), eta | 6p21.1 | Xeroderma pigmentosa V | Squamous cell cancer (skin) | autosomal recessive |
| *PRKAR1A* | protein kinase, cAMP-dependent, regulatory, type I, alpha | 17q23-q24 | Carney complex | Myxoma (cardiac/cutaneous/breast) Thyroid cancer Sex cord-stromal tumor | autosomal dominant |
| *PRSS1* | protease, serine, 1 (trypsin 1) | 7q34 |  | Pancreatic cancer | autosomal dominant |
| *PTCH1* | patched 1 | 9q22.3 | Nevoid basal cell carcinoma syndrome Gorlin Syndrome | Basal cell carcinoma Medulloblastoma | autosomal dominant |
| *PTEN* | phosphatase and tensin homolog | 10q23.3 | Cowden Syndrome PTEN hamartoma tumor syndrome | Breast cancer Thyroid cancer Endometrial cancer | autosomal dominant |
| *PTPN11* | protein tyrosine phosphatase, non-receptor type 11 | 12q24.13 | Noonan syndrome | JMML neuroblastoma | autosomal dominant |
| *RAD51C* | RAD51 homolog C (S. cerevisiae) | 17q25.1 | Fanconi anaemia (O) (biallelic mutations) | Monoallelic mutations:  Ovarian cancer | autosomal recessive  autosomal dominant |
| *RAD51D* | RAD51 homolog D (S. cerevisiae) | 17q11 |  | Ovarian cancer | autosomal dominant |
| *RB1* | retinoblastoma 1 | 13q14.2 |  | Retinoblastoma Pinealoma Sarcoma Melanoma | autosomal dominant |
| *RECQL4* | RecQ protein-like 4 | 8q24.3 | Rothmund-Thompson syndrome | Osteosarcoma Basal cell carcinoma Squamous cell carcinoma | autosomal recessive |
| *RET* | ret proto-oncogene | 10q11.2 | Multiple endocrine neoplasia 2A/2B  Familial medullary thyroid carcinoma | Medullary thyroid cancer Pheochromocytoma | autosomal dominant |
| *RHBDF2* | rhomboid 5 homolog 2 (Drosophila) | 17q25.1 |  | Esophageal cancer | autosomal dominant |
| *RMRP* | RNA component of mitochondrial RNA processing endoribonuclease | 9p13.3 | Cartilage-hair hypoplasia syndrome | Non-hodgkin lymphoma Squamous carcinoma (bcc) Leukemia | autosomal recessive |
| *RUNX1* | runt-related transcription factor 1 | 21q22.3 |  | Myeloid hematological malignancy (leukemia) | autosomal dominant |
| *SBDS* | Shwachman-Bodian-Diamond syndrome | 7q11 | Schwachman-Diamond syndrome | Myeloid hematological malignancy | autosomal recessive |
| *SDHA* | succinate dehydrogenase complex, subunit A, flavoprotein (Fp) | 5p15.33 | Carney-Stratakis syndrome | Paraganglioma Pheochromocytoma Gastrointestinal stromal tumor (GIST) | autosomal recessive  autosomal dominant |
| *SDHAF2* | succinate dehydrogenase complex assembly factor 2 | 11q12.2 | Familial paraganglioma-pheochromocytoma syndrome | Paraganglioma Pheochromocytoma | autosomal dominant |
| *SDHB* | succinate dehydrogenase complex, subunit B, iron sulfur (Ip) | 1p36.1-p35 | Familial paraganglioma-pheochromocytoma syndrome | Paraganglioma Pheochromocytoma Renal cell cancer | autosomal dominant |
| *SDHC* | succinate dehydrogenase complex, subunit C, integral membrane protein, 15kDa | 1q21 | Familial paraganglioma-pheochromocytoma syndrome | Paraganglioma Pheochromocytoma Gastrointestinal stromal tumor (GIST) | autosomal dominant |
| *SDHD* | succinate dehydrogenase complex, subunit D, integral membrane protein | 11q23 | Familial paraganglioma-pheochromocytoma syndrome | Paraganglioma Pheochromocytoma Gastrointestinal stromal tumor (GIST) | autosomal dominant |
| *SERPINA1* | serpin peptidase inhibitor, clade A (alpha-1 antiproteinase, antitrypsin), member 1 | 14q32.13 | Alpha1 antitrypsin defiency | Hepatocellular carcinoma | autosomal recessive |
| *SH2D1A* | SH2 domain containing 1A | Xq25 | Lymphoproliferative disease | Lymphoma | X-linked recessive |
| *SLC25A13* | solute carrier family 25 (aspartate/glutamate carrier), member 13 | 7q21.3 | Citrullinaemia | Hepatocellular carcinoma | autosomal recessive |
| *SMAD4* | SMAD family member 4 | 18q21.1 | Juvenile polyposis syndrome | Colorectal cancer | autosomal dominant |
| *SMARCA4* | SWI/SNF related, matrix associated, actin dependent regulator of chromatin, subfamily a, member 4 | 19p13.2 | Rhabdoid predisposition syndrome | Rhabdoid tumor | autosomal dominant |
| *SMARCB1* | SWI/SNF related, matrix associated, actin dependent regulator of chromatin, subfamily b, member 1 | 22q11 | Rhabdoid predisposition syndrome | Rhabdoid tumor (renal, extra-renal) Central primitive neuroectodermal tumor | autosomal dominant |
| *SMARCE1* | SWI/SNF related, matrix associated, actin dependent regulator of chromatin, subfamily e, member 1 | 17q21.2 |  | Mengingioma | autosomal dominant |
| *SOS1* | son of sevenless homolog 1 (Drosophila) | 2p22.1 | Noonan syndrome | Rhabdomyosarcoma | autosomal dominant |
| *SRY* | sex determining region Y | Yp11.31 |  | Gonadoblastoma | Y-linked |
| *STAT3* | signal transducer and activator of transcription 3 (acute-phase response factor) | 17q21.1 | Hyper-immunoglobulin E syndrome | Lymphoma | autosomal dominant |
| *STK11* | serine/threonine kinase 11 | 19p13.3 | Peutz-Jeghers syndrome | Colorectal cancer Gastric cancer Breast cancer Sex cord-stromal tumor | autosomal dominant |
| *SUFU* | suppressor of fused homolog (Drosophila) | 10q24.32 |  | Medulloblastoma, meningioma | autosomal dominant |
| *TERT* | telomerase reverse transcriptase | 5p15.33 | Dyskeratosis congenita | acute myeloid leukemia  Squamous cell carcinoma (head + neck, anorectal)  Melanoma | autosomal recessive  autosomal dominant |
| *TGFBR1* | transforming growth factor, beta receptor 1 | 9q22.33 | Multiple self-healing squamous epithelioma (MSSE) Ferguson-Smith syndrome | Squamous cell carcinoma (skin) | autosomal dominant |
| *TMEM127* | transmembrane protein 127 | 2q11.2 |  | Pheochromocytoma | autosomal dominant |
| *TNFRSF6 (FAS)* | transforming growth factor, beta receptor 1 | 10q23.31 | Autoimmunie lymphoproliferative syndrome | Lymphoma | autosomal dominant |
| *TP53* | tumor protein p53 | 17p13.1 | Li-Fraumeni syndrome | Breast cancer Sarcoma Adrenocortical carcinoma Astrocytoma | autosomal dominant |
| *TRIM37* | tripartite motif containing 37 | 17q22 | Mulibrey-nanism | Wilms tumor | autosomal recessive |
| *TSC1* | tuberous sclerosis 1 | 9q34 | Tuberous sclerosis 1 | Renal cell cancer, angiomyolipoma Subependymal giant cell astrocytoma Rhabdomyoma (cardiac) | autosomal dominant |
| *TSC2* | tuberous sclerosis 2 | 16p13.3 | Tuberous sclerosis 2 | Renal cell cancer, angiomyolipoma Subependymal giant cell astrocytoma Rhabdomyoma (cardiac) | autosomal dominant |
| *UROD* | uroporphyrinogen decarboxylase | 1p34.1 | Porphyria (cutanea tarda) | hepatocellular carcinoma | autosomal recessive  autosomal dominant |
| *VHL* | von Hippel-Lindau tumor suppressor, E3 ubiquitin protein ligase | 3p25 | Von Hippel-Lindau syndrome | Renal cell cancer Pheochromocytoma Neuroendocrine tumor (pancreas) Hemangioblastoma (central nervous system, retina) | autosomal dominant |
| *WAS* | Wiskott-Aldrich syndrome | Xp11.23 | Wiskott-Aldrich syndrome WAS-related syndrome | Lymphoma | X-linked recessive |
| *WRN* | Werner syndrome, RecQ helicase-like | 8p12 | Werner syndrome | Sarcoma Melanoma Thyroid cancer | autosomal recessive |
| *WT1* | Wilms tumor 1 | 11p13 | WAGR syndrome Denys-Drash syndrome Frasier syndrome | Wilms tumor Gonadoblastoma | autosomal dominant |
| *XPA* | xeroderma pigmentosum, complementation group A | 9q22.3 | Xeroderma pigmentosum (A) | Basal cell carcinoma Squamous cell carcinoma Melanoma | autosomal recessive |
| *XPC* | xeroderma pigmentosum, complementation group C | 3p25 | Xeroderma pigmentosum (C) | Basal cell carcinoma Squamous cell carcinoma Melanoma | autosomal recessive |

**Supplementary Table 2: Details of mutations in all CSGs**

| **Gene** | **Chr** | **Position** | **Ref** | **Alt** | **Impact** | **Case/Control^$^** | **Clinvar** | **Family history*** |  |  |
| --- | --- | --- | --- | --- | --- | --- | --- | --- | --- | --- |
| *ABCB11* | 2 | 169847329 | T | C | M | 1 | P |  |  |  |
| *ABCB11* | 2 | 169847329 | T | C | M | 1 | P |  |  |  |
| *ATM* | 11 | 108115639 | CT | C | FS | 1 | P |  |  |  |
| *ATM* | 11 | 108143258 | G | A | SA | 1 | - |  |  |  |
| *ATM* | 11 | 108155007 | AG | A | FS | 2 | P | father(1539/155) |  |  |
| *ATM* | 11 | 108155007 | AG | A | FS | 1 | P |  |  |  |
| *ATM* | 11 | 108155138 | C | T | SG | 2 | P | father(1539) |  |  |
| *ATM* | 11 | 108163434 | T | TAGTA | FS | 1 | - |  |  |  |
| *ATM* | 11 | 108196144 | G | A | M | 1 | LP;P |  |  |  |
| *ATM* | 11 | 108198392 | T | TA | FS | 1 | P |  |  |  |
| *ATM* | 11 | 108202286 | T | C | SD | 2 | LP | father(1539)sister(174) | |  |
| *ATM* | 11 | 108202611 | CTCTAGAATT | C | ID | 2 | P | father(162)sister(157) | |  |
| *ATM* | 11 | 108235831 | TTGAC | T | FS | 1 | LP |  |  |  |
| *ATM* | 11 | 108235935 | C | T | SG | 1 | LP;P |  |  |  |
| *BLM* | 15 | 91298161 | CTG | C | FS | 2 | - | father(1539)mother(203) | |  |
| *BLM* | 15 | 91306246 | C | T | SG | 2 | - | father(1539/185)mother(174) | |  |
| *BLM* | 15 | 91328183 | C | T | SG | 1 | LP;P |  |  |  |
| *BLM* | 15 | 91328183 | C | T | SG | 2 | LP;P | father(1539/185/155) | |  |
| *BRCA1* | 17 | 41215920 | G | T | M | 2 | P | sister(174)father(1539)mother(183) | | |
| *BRCA1* | 17 | 41215948 | G | A | M | 1 | P |  |  |  |
| *BRCA1* | 17 | 41243788 | TAGAC | T | FS | 1 | LP;P |  |  |  |
| *BRCA1* | 17 | 41243788 | TAGAC | T | FS | 2 | LP;P | sister(1539/174) |  |  |
| *BRCA1* | 17 | 41243788 | TAGAC | T | FS | 2 | LP;P | father(185)mother(1539) | |  |
| *BRCA1* | 17 | 41244318 | CCT | C | FS | 2 | LP;P | father(1539/155)mother(174/183/156) | | |
| *BRCA1* | 17 | 41245354 | C | A | SG | 1 | P |  |  |  |
| *BRCA1* | 17 | 41245697 | GGT | G | FS | 1 | - |  |  |  |
| *BRCA1* | 17 | 41256939 | CA | C | FS | 1 | - |  |  |  |
| *BRCA2* | 13 | 32911650 | T | G | SG | 1 | P |  |  |  |
| *BRCA2* | 13 | 32912171 | CTG | C | FS | 2 | P | father(153/151/157)sister(153/183) | | |
| *BRCA2* | 13 | 32912180 | TC | T | FS | 2 | P | father(1539/155)mother(174/183/156) | | |
| *BRCA2* | 13 | 32914174 | C | A | SG | 2 | P | mother(1539) |  |  |
| *BRCA2* | 13 | 32914437 | GT | G | FS | 1 | P;RF |  |  |  |
| *BRCA2* | 13 | 32914766 | CTT | C | FS | 2 | P | father(1539)mother(174) | |  |
| *BRCA2* | 13 | 32915027 | G | GA | FS | 2 | - | father(1539)mother(1539) | |  |
| *BRCA2* | 13 | 32953986 | GTA | G | FS | 1 | P |  |  |  |
| *BRIP1* | 17 | 59761411 | CTCTT | C | FS | 1 | P |  |  |  |
| *BRIP1* | 17 | 59761413 | CTTTG | C | FS | 2 | - | father(1539) |  |  |
| *BRIP1* | 17 | 59793309 | T | TA | SR | 2 | LP;US | brother(153) |  |  |
| *BRIP1* | 17 | 59793412 | G | A | SG | 1 | P |  |  |  |
| *BRIP1* | 17 | 59793412 | G | A | SG | 2 | P | brother(153)father(153)mother(179) | | |
| *BRIP1* | 17 | 59821793 | CTT | C | FS | 2 | P | sister(174/182)brother(1539)mother(1539) | | |
| *BRIP1* | 17 | 59821793 | CTT | C | FS | 2 | P | father(1539) |  |  |
| *BRIP1* | 17 | 59853846 | CT | C | FS | 1 | - |  |  |  |
| *BRIP1* | 17 | 59937223 | G | C | M | 1 | LP;P;US |  |  |  |
| *BRIP1* | 17 | 59937223 | G | C | M | 2 | LP;P;US | mother(172/174/155/153/151) | |  |
| *BRIP1* | 17 | 59937230 | CAACA | C | FS | 1 | - |  |  |  |
| *BUB1B* | 15 | 40501902 | T | G | SG | 2 | - | father(1539) |  |  |
| *CDC73* | 1 | 193181597 | C | T | SG | 1 | - |  |  |  |
| *CDK4* | 12 | 58144443 | GA | G | FS | 1 | - |  |  |  |
| *CHEK2* | 22 | 29106021 | TTC | T | FS | 1 | - |  |  |  |
| *CHEK2* | 22 | 29106023 | C | A | SG | 1 | - |  |  |  |
| *CHEK2* | 22 | 29107974 | C | T | M | 2 | P;US | father(1539) |  |  |
| *CHEK2* | 22 | 29121015 | C | T | M | 1 | P;US |  |  |  |
| *CHEK2* | 22 | 29121242 | G | A | M | 1 | LP;P |  |  |  |
| *COL7A1* | 3 | 48602832 | GCTGTCCTCACCTTC | G | SD | 2 | - | father(153)mother(173) | |  |
| *COL7A1* | 3 | 48628154 | G | A | SG | 1 | - |  |  |  |
| *COL7A1* | 3 | 48628154 | G | A | SG | 1 | - |  |  |  |
| *COL7A1* | 3 | 48630971 | T | C | M | 1 | P |  |  |  |
| *CYLD* | 16 | 50783850 | GT | G | FS | 2 | - | father(1539) |  |  |
| *DDB2* | 11 | 47256397 | TCAAA | T | FS | 2 | - | father(1539)daughter(1539) | |  |
| *DICER1* | 14 | 95572101 | G | A | SG | 1 | - |  |  |  |
| *ERCC2* | 19 | 45855519 | C | T | M | 1 | P |  |  |  |
| *ERCC2* | 19 | 45856344 | AG | A | FS | 2 | - | father(185)mother(1539) | |  |
| *ERCC2* | 19 | 45860626 | G | C | M | 1 | P |  |  |  |
| *ERCC2* | 19 | 45860626 | G | C | M | 1 | P |  |  |  |
| *ERCC2* | 19 | 45860626 | G | C | M | 2 | P | sister(162)father(162)mother(153) | | |
| *ERCC3* | 2 | 128030510 | CT | C | FS | 2 | - | sister(174)father(1539) | |  |
| *ERCC3* | 2 | 128030510 | CT | C | FS | 2 | - | sister(153)mother(153) | |  |
| *ERCC3* | 2 | 128038128 | A | AT | FS | 2 | - | mother(1539) |  |  |
| *ERCC3* | 2 | 128050332 | G | A | SG | 1 | - |  |  |  |
| *ERCC4* | 16 | 14020487 | G | A | M | 2 | P | daughter(180)sister(153)father(159) | | |
| *ERCC4* | 16 | 14029187 | CA | C | FS | 2 | - | father(1539)sister(172) | |  |
| *ERCC4* | 16 | 14041848 | C | T | M | 1 | P |  |  |  |
| *ERCC4* | 16 | 14041848 | C | T | M | 2 | P | father(1539)mother(1539) | |  |
| *ERCC4* | 16 | 14041848 | C | T | M | 2 | P | brother(1539)sister(199) | |  |
| *ERCC4* | 16 | 14041848 | C | T | M | 1 | P |  |  |  |
| *ERCC5* | 13 | 103514706 | G | GTGTGTGC | FS | 2 | - | father(1539)sister(174)mother(174) | | |
| *EXT2* | 11 | 44253924 | CGGTT | C | FS | 2 | - | father(1539/155) |  |  |
| *FAH* | 15 | 80452206 | G | T | SG | 1 | - |  |  |  |
| *FAH* | 15 | 80460605 | G | T | SA | 2 | P | father(153)mother(153/172/162) | | |
| *FAH* | 15 | 80460605 | G | T | SA | 1 | P |  |  |  |
| *FAH* | 15 | 80460605 | G | T | SA | 1 | P |  |  |  |
| *FAH* | 15 | 80473411 | G | T | SG | 1 | P |  |  |  |
| *FAH* | 15 | 80473411 | G | T | SG | 1 | P |  |  |  |
| *FANCA* | 16 | 89807249 | GAGA | G | ID | 1 | P |  |  |  |
| *FANCA* | 16 | 89858441 | GCCAA | G | FS | 1 | P |  |  |  |
| *FANCA* | 16 | 89858441 | GCCAA | G | FS | 1 | P |  |  |  |
| *FANCC* | 9 | 97864024 | G | A | SG | 1 | P |  |  |  |
| *FANCC* | 9 | 97912338 | G | A | SG | 1 | P |  |  |  |
| *FANCC* | 9 | 97912338 | G | A | SG | 2 | P | mother(1539) |  |  |
| *FANCC* | 9 | 97933391 | TTC | T | FS | 1 | P |  |  |  |
| *FANCC* | 9 | 97933391 | TTC | T | FS | 1 | P |  |  |  |
| *FANCC* | 9 | 98011506 | TC | T | FS | 2 | P | father(153/208) |  |  |
| *FANCG* | 9 | 35074486 | G | A | SG | 1 | - |  |  |  |
| *FANCG* | 9 | 35074486 | G | A | SG | 2 | - | father(1539) |  |  |
| *FH* | 1 | 241665871 | C | T | SA | 1 | - |  |  |  |
| *FLCN* | 17 | 17118498 | C | T | SD | 2 | - | father(1539) |  |  |
| *FLCN* | 17 | 17120402 | G | C | SG | 2 | - | father(1539) |  |  |
| *FLCN* | 17 | 17122501 | GCTTT | G | FS | 2 | P | mother(1539) |  |  |
| *GBA* | 1 | 155209430 | A | C | M | 1 | LP |  |  |  |
| *HMBS* | 11 | 118962124 | G | A | M | 1 | P;US |  |  |  |
| *ITK* | 5 | 156675910 | C | T | SG | 1 | - |  |  |  |
| *MEN1* | 11 | 64573702 | A | G | SD | 2 | - | brother(1539/199)father(1539/172) | | |
| *NBN* | 8 | 90960063 | T | A | SG | 1 | P |  |  |  |
| *NBN* | 8 | 90971084 | T | C | SA | 1 | - |  |  |  |
| *NBN* | 8 | 90983401 | CTGTT | C | FS | 2 | P | father(1539)mother(151) | |  |
| *NBN* | 8 | 90983441 | ATTTGT | A | FS | 2 | P;RF | father(1539)daughter(208)brother(162) | | |
| *NF1* | 17 | 29557390 | A | G | M | 2 | P | father(1539) |  |  |
| *PALB2* | 16 | 23614792 | G | C | SG | 2 | P;RF | father(1539)sister(172) | |  |
| *PALB2* | 16 | 23625409 | AT | A | FS | 1 | P;RF |  |  |  |
| *POLH* | 6 | 43573048 | C | T | SG | 2 | - | father(1539) |  |  |
| *PTPN11* | 12 | 112891120 | C | T | M | 1 | LP |  |  |  |
| *RAD51C* | 17 | 56770101 | C | T | SG | 1 | P |  |  |  |
| *RAD51C* | 17 | 56801435 | AATCTTTC | A | FS | 1 | - |  |  |  |
| *RAD51C* | 17 | 56809908 | CAGT | C | SR | 1 | LP;US |  |  |  |
| *RAD51D* | 17 | 33433425 | G | A | SG | 2 | P;RF | father(1539) |  |  |
| *RAD51D* | 17 | 33433425 | G | A | SG | 1 | P;RF |  |  |  |
| *RAD51D* | 17 | 33434009 | G | A | SG | 2 | - | sister(1541)mother(199) | |  |
| *RECQL4* | 8 | 145737689 | CCT | C | FS | 1 | - |  |  |  |
| *RECQL4* | 8 | 145739831 | G | A | SG | 2 | - | father(153) |  |  |
| *RECQL4* | 8 | 145740366 | CA | C | FS | 2 | LP;P | father(1539) |  |  |
| *RECQL4* | 8 | 145740366 | CA | C | FS | 1 | LP;P |  |  |  |
| *RECQL4* | 8 | 145740366 | CA | C | FS | 1 | LP;P |  |  |  |
| *RECQL4* | 8 | 145740366 | CA | C | FS | 1 | LP;P |  |  |  |
| *RECQL4* | 8 | 145740366 | CA | C | FS | 1 | LP;P |  |  |  |
| *RECQL4* | 8 | 145740366 | CA | C | FS | 2 | LP;P | father(1539)mother(190) | |  |
| *RECQL4* | 8 | 145740627 | C | T | SA | 1 | P |  |  |  |
| *RET* | 10 | 43622171 | G | A | SD | 1 | - |  |  |  |
| *SDHA* | 5 | 223624 | C | T | SG | 1 | LP;P |  |  |  |
| *SDHA* | 5 | 223624 | C | T | SG | 1 | LP;P |  |  |  |
| *SDHB* | 1 | 17350520 | G | C | M | 1 | LP;P |  |  |  |
| *SDHD* | 11 | 112041345 | G | C | SA | 1 | - |  |  |  |
| *SERPINA1* | 14 | 94847286 | T | A | M | 1 | LP;O |  |  |  |
| *SERPINA1* | 14 | 94847380 | C | T | M | 1 | LP |  |  |  |
| *SERPINA1* | 14 | 94847404 | T | A | SG | 2 | LP;O | father(1539)daughter(1539) | |  |
| *SLC25A13* | 7 | 95799438 | C | T | SA | 1 | - |  |  |  |
| *SLC25A13* | 7 | 95822414 | G | A | SG | 2 | P | father(185)mother(1539) | |  |
| *SLC25A13* | 7 | 95906521 | G | A | SG | 1 | - |  |  |  |
| *TGFBR1* | 9 | 101907018 | GC | G | FS | 2 | - | father(1539) |  |  |
| *TP53* | 17 | 7579485 | C | A | SG | 2 | - | father(162)mother(1539) | |  |
| *TRIM37* | 17 | 57134354 | G | A | SG | 1 | - |  |  |  |
| *TRIM37* | 17 | 57153076 | C | T | SA | 2 | - | father(1539)mother(162/191) | |  |
| *VHL* | 3 | 10191581 | C | T | M | 1 | P |  |  |  |
| *VHL* | 3 | 10191581 | C | T | M | 1 | P |  |  |  |
| *WRN* | 8 | 30921804 | G | A | SA | 1 | - |  |  |  |
| *WRN* | 8 | 30938648 | C | T | SG | 1 | P |  |  |  |
| *WRN* | 8 | 30948459 | G | A | SD | 1 | - |  |  |  |
| *WRN* | 8 | 30982042 | CT | C | FS | 1 | - |  |  |  |
| *XPA* | 9 | 100451865 | CCA | C | FS | 1 | - |  |  |  |

^$^1=control, 2=case

***ICD Codes**

1539 Colon, unspecified (large intestine NOS)

155 Malignant neoplasm of liver and intrahepatic bile ducts

174 Malignant neoplasm of female breast

157 Malignant neoplasm of pancreas

203 Multiple myeloma and immunoproliferative neoplasms

185 Malignant neoplasm of prostate

183 Malignant neoplasm of ovary and other uterine adnexa

156 Malignant neoplasm of gallbladder and extrahepatic bile ducts

153 Malignant neoplasm of colon

179 Malignant neoplasm of uterus, part unspecified

182 Malignant neoplasm of body of uterus

172 Malignant melanoma of skin

151 Malignant neoplasm of stomach

173 Other malignant neoplasm of skin

162 Malignant neoplasm of trachea, bronchus and lung

180 Malignant neoplasm of cervix uteri

159 Malignant neoplasm of other and ill-defined sites within the digestive organs and peritoneum

199 Malignant neoplasm without specification of site

208 Leukaemia of unspecified cell type

1541 Rectum (rectal ampulla)

191 Malignant neoplasm of brain
